# Atavism

**Published:** 1883-04

**Authors:** Hubbard W. Mitchell

**Affiliations:** Professor of Comparative Anatomy in the Columbia Veterinary College


### Art. XIII—ATAVISM.

BY HUBBARD W. MITCHELL, M.D.,

*Professor of Comparative Anatomy in the Columbia Veterinary College.*

BY the the term Atavism we mean that strange and peculiar power or tendency of plants and animals to revert to and acquire certain latent or long lost characters belonging to their ancestors. The word is derived from the Latin, *Atavus*, an ancestor.

But little is known of the real nature of this tendency, although we see numerous examples of it in the animal and vegetable world.

The importance of this subject is very great, especially to breeders of pure blooded horses and cattle, as well as to those who delight to rear fine dogs and fowls. Every little while we see, in pure strains of animals who have been bred for long periods of time with the utmost care, certain new and strange qualities appear which cannot be accounted for, and which are attributed to some mistake or accident of a cross with an inferior breed. This appearance of strange qualities is due to no accident, but is a *reversion* or *turning back* of the animal's nature to some trait or quality away back in the ancestral line, and is an instance of true *Atavism.* Certain characters lie dormant for considerable lengths of time, often for many generations, and then suddenly reappear. The late Mr. Darwin wrote upon this subject, and was, so far as I am aware, the first to call attention to it. In this country the word Atavism has been seldom used, and then only in a vague and indefinite way, because its meaning was not well understood. In order to understand the principle of Atavism, it is necessary to know something of the characters and habits of certain species of animals in their feral state that have originally been derived from a common stock. After the early geological horses with five toes had been replaced by modern horses with one toe (the others becoming rudimentary), these animals in the feral state were often found to have certain shoulder and leg stripes, and hair markings on different parts of the body. In the later processes of high breeding from horses selected with unusual care, and for a long series of years, with the especial view of retaining, and perfecting, and transmitting certain valuable qualities, as of color, form, speed; suddenly, and for no *apparent* reason, a shoulder stripe, or a leg stripe, or a patch or color, or a thick leg, or a coarse skull has appeared to the utter astonishment, of course, of the breeder. The reason of this is that the animal has *reverted* to the condition of the original horse having these peculiar qualities, through some subtle tendency which is not yet completely understood, and which we call Atavism. When tame horses have been turned loose in parts of Europe, Asia and South America, they have, in process of time, reverted to the wild state, and have assumed the duller colors and hair markings of the true wild horse. Instances, more curious still, have been recorded where horses have developed the rudimentary meta-tarsal and meta-carpal bones, thus reverting to the original five-toed state. These bones have developed into what are known as *supernumary digits.* We see cases, frequently, of supernumary digits in many animals and man.

The original Dog was a carnivorous scavenger. The great variety of breeds we see at the present time have all sprung from selection and crossing, continued through long periods of time. In spite of this lapse of time, and the changes due to breeding, we see traits that have been transmitted from the feral state, and that have never been extinguished, as, for example, the fondness of dogs for carrion. They delight to roll their bodies upon it, enjoying the putrid odor, and finally devouring it with fierce avidity. Also the habit of turning round and round before lying down, and of scratching to cover their excrement. This habit may still be observed in wild animals of the dog family, as the Fox, Wolf and Hyæna. Its permanence is curious and interesting, as showing how so simple a trait can be so long transmitted unchanged by domestication. The highly bred and artificially cared for fancy dogs, who have been the objects of especial care by breeders, for several generations will suddenly manifest their love of carrion, thus showing how strong the tendency is to reversion to their original feral state.

This tendency is very strong in Pigs. These animals have shown a remarkable degree of plasticity of form under the wise hand of selection and domestication. Instances are noted where single members of fine white breeds, with small teeth and rounded bodies, have suddenly assumed the brown color and the formidable tusk of the Wild Boar. This can only be accounted for on the principle of Atavism. In the Ozark Mountains of South-western Missouri are great numbers of pigs that have actually reverted to the feral state. In a few generations they have assumed all the characters of their original wild progenitors, the great ferocity of disposition, the brown color, the coarse bristles, the lank body, and the dangerous tusk. They feed upon the acorns from the scrub oaks of that region, and when seen among the scattered trees which give that locality its strange and lonely park-like appearance, they remind one of the typical wild boar of German legend.

Atavism is not unfrequently observed in Sheep. In the original state these ruminating mammals were of darker color, and coarser hair or wool. They were derived from the same common stock as the goat, llama, camel and giraffe. The close wool is due to a colder climate. Its fineness is the result of selected breeding, as also its whiteness. Instances are recorded where a ewe has produced white lambs for a number of years, and then a black one, or one with hairy wool, and no reason could be assigned for it. In such cases some remote ancestor has had black wool, or coarse, hairy, goat-like wool, and this has reappeared after many years of close and careful breeding, notwithstanding the fact that neither the mother, nor several generations behind her, had ever shown the slightest trace of such taint. This can only be due to the principle of Atavism. The tendency to reversion is especially strong in the Felidæ, particularly in the domestic cat. This animal seems to have stored up within itself all the latent characters of its original ferocity, and eagerness and cruelty of disposition, ready upon slight neglect of domestication to break out and carry the animal swiftly back to the feral state. The power of man has been exerted so slightly upon these animals that it may be said that the step of reversion to the wild state is an extremely short one.

Many instances could be adduced of reversion in the Rabbit, in the Pigeon and in the Fowl. These animals are extremely plastic in their nature under the hand of man. The color of the rabbit, and even its form, responds readily to the influences of selected breeding and domestication. The pigeon responds more readily still, and it is a comparatively easy matter to shorten the beak or change the plumage, or to breed tumblers or pouters. These last characters are very unstable and evanescent, and due, it is believed, to some injury, originally, to its *cerebellum.* Breeders have found it difficult to maintain the habit of tumbling, and if their care in pairing them is relaxed the birds revert quickly to their original state.

The light Brahma fowl, after being bred for a long time with the especial view of keeping the strain pure, will often show feathers of a different color, and when the line of ancestry is traced back, it has been discovered that some remote progenitor had been similarly colored, and this taint of color has reappeared as a break in a long line of pure offspring. This is clearly due to Atavism.

There are instances of Atavism in the human species. A friend in Louisiana related to me the case of a white woman who many years ago became pregnant by a negro. Her child was of course a mulatto. Subsequently she married a white man, and was the mother of six or seven perfectly white children. She finally gave birth to an eighth child, and this one was a dark mulatto, with thick lips and curly wool, to the utter surprise of her husband and friends. The white man was the father of this, as of the others. This case is vouched for.

Atavism also is seen in plants, in flowers, as well as in fruits. I will not, however, give any instances of this. I have given no authorities for any of the foregoing cases. They exist, however, and are accessible if wanted. I have preferred to give the instances simply, and with the view of calling the attention of others to this interesting subject. It is impossible, in the present state of our knowledge, to explain what this influence or tendency is that causes certain long lost characters to reappear at intervals in animals. That it does exist is attested beyond doubt by breeders and careful observers. So strange a circumstance at first excited much surprise, and the owners of animals thus reverting were inclined to think that an accidental cross was the reason. Close study, however, and long continued observation revealed the fact that there was a curious tendency in animals to revert to original types or characters, and this tendency has received the scientific appellation of Atavism.

